# Leveraging citizen science for monitoring urban forageable plants

**DOI:** 10.1093/gigascience/giae007

**Published:** 2024-03-05

**Authors:** Filipi Miranda Soares, Luís Ferreira Pires, Maria Carolina Garcia, Yamine Bouzembrak, Lidio Coradin, Natalia Pirani Ghilardi-Lopes, Rubens Rangel Silva, Aline Martins de Carvalho, Benildes Coura Moreira dos Santos Maculan, Sheina Koffler, Uiara Bandineli Montedo, Debora Pignatari Drucker, Raquel Santiago, Anand Gavai, Maria Clara Peres de Carvalho, Ana Carolina da Silva Lima, Hillary Dandara Elias Gabriel, Stephanie Gabriele Mendonça de França, Karoline Reis de Almeida, Bárbara Junqueira dos Santos, Antonio Mauro Saraiva

**Affiliations:** Escola Politécnica, Universidade de São Paulo, São Paulo, SP 05508-010, Brazil; Faculty of Electrical Engineering, Mathematics and Computer Science, University of Twente, Enschede, 7522 NB, The Netherlands; Faculty of Electrical Engineering, Mathematics and Computer Science, University of Twente, Enschede, 7522 NB, The Netherlands; Programa de Pós Graduação em Arquitetura, Urbanismo e Design, Centro Universitário Belas Artes de São Paulo, São Paulo, SP, 04018-010, Brazil; Information Technology Group, Wageningen University and Research, Wageningen, 6706 KN, The Netherlands; Plants for the Future Project, Brasília, DF, 70772-090, Brazil; Centro de Ciências Naturais e Humanas, Universidade Federal do ABC, São Bernardo do Campo, SP 09606-045, Brazil; Centro Universitário Una, Belo Horizonte, MG 30160-011, Brazil; Departamento de Nutrição, Faculdade de Saúde Pública, Universidade de São Paulo, São Paulo, SP 01246-904, Brazil; Programa de Pós-Graduação em Gestão e Organização do Conhecimento, Universidade Federal de Minas Gerais, Belo Horizonte, MG 31270-901, Brazil; Instituto de Estudos Avançados, Universidade de São Paulo, São Paulo, SP 05508-060, Brazil; Escola Politécnica, Universidade de São Paulo, São Paulo, SP 05508-010, Brazil; Embrapa Agricultura Digital, Campinas, SP 13083-886, Brazil; Faculdade de Nutrição, Universidade Federal de Goiás, Goiânia, GO 74605-080, Brazil; Faculty of Behavioural, Management and Social Sciences (BMS), Industrial Engineering and Business Information Systems (IEBIS), Enschede, 7522 NB, the Netherlands; Escola de Artes, Ciências e Humanidades, Universidade de São Paulo, São Paulo, SP 03828-000, Brazil; Escola de Ciências da Informação, Universidade Federal de Minas Gerais, Belo Horizonte, MG 31270-901, Brazil; Escola Politécnica, Universidade de São Paulo, São Paulo, SP 05508-010, Brazil; Escola Politécnica, Universidade de São Paulo, São Paulo, SP 05508-010, Brazil; Escola Politécnica, Universidade de São Paulo, São Paulo, SP 05508-010, Brazil; Instituto de Pesquisas Energéticas e Nucleares, Universidade de São Paulo, São Paulo, SP 05508-000, Brazil; Escola Politécnica, Universidade de São Paulo, São Paulo, SP 05508-010, Brazil; Instituto de Estudos Avançados, Universidade de São Paulo, São Paulo, SP 05508-060, Brazil

**Keywords:** fruit-bearing plants, urban foraging, wild food, urban food trees, food forest, fruit tree

## Abstract

Urbanization brings forth social challenges in emerging countries such as Brazil, encompassing food scarcity, health deterioration, air pollution, and biodiversity loss. Despite this, urban areas like the city of São Paulo still boast ample green spaces, offering opportunities for nature appreciation and conservation, enhancing city resilience and livability. Citizen science is a collaborative endeavor between professional scientists and nonprofessional scientists in scientific research that may help to understand the dynamics of urban ecosystems. We believe citizen science has the potential to promote human and nature connection in urban areas and provide useful data on urban biodiversity.

## Background

In the dynamic landscapes of urban environments, the intricate tapestry of biodiversity is often overlooked in the midst of concrete and steel. However, an emerging force is transforming the way we perceive and comprehend the ecological fabric of cities—citizen science (CS). This commentary delves into the pivotal role of CS in monitoring urban biodiversity, unearthing its profound implications for understanding, conserving, and elevating the intricate life forms that coexist within our urban sprawls.

As urbanization continues to reshape the world, a robust understanding of the ecological dynamics within cities is indispensable for harmonizing human progress with environmental preservation. The engagement of citizen scientists emerges as an ingenious solution to this challenge.

## Citizen science for urban biodiversity monitoring

CS initiatives encompass distinct levels of public participation, from collecting data to creating new research questions and projects [1]. In general, most CS projects are contributory, relying on public participation mainly for data collection. CS thus allows the creation of large datasets while approximating the public to the scientific process and providing new learning opportunities [1].

In the field of life sciences, especially in ecology and biodiversity, applications such as eBird (https://ebird.org/home), Pl@ntNet (https://identify.plantnet.org), and iNaturalist (https://www.inaturalist.org/) stand out for both their number of users worldwide and the volume of data collected. eBird and Pl@ntnet cover specific taxonomic groups, while iNaturalist includes all life forms.

The data available on iNaturalist can be leveraged in monitoring urban biodiversity. While some studies have utilized iNaturalist for this purpose (e.g., [2–4]), there is limited research on plant diversity and distribution using data from this platform, such as [5]. Fruit-bearing plants constitute a pivotal group of organisms crucial to the functionality of urban ecosystems, owing to their capacity to deliver an array of provisioning services. In light of this perspective, the Pomar Urbano (Urban Orchard) initiative serves as a collaborative platform, uniting researchers and citizen scientists across Brazil to monitor forageable plants within urban landscapes comprehensively.

iNaturalist allows managing observations of interest within projects like Pomar Urban*o*, referred to as iNaturalist Projects. These projects come in 3 types: collection projects, umbrella projects, and traditional projects (https://www.inaturalist.org/pages/managing-projects). Pomar Urbano makes use of umbrella and collection projects. Observations posted to iNaturalist by any user are included if they (i) pertain to a plant species listed in the project and (ii) are located in one of the capitals of the 27 Brazilian federative units [6]. Each capital has its collection project. The umbrella project then aggregates data from all 27 individual collection projects. Pomar Urbano data can be accessed via iNaturalist (https://www.inaturalist.org/projects/pomar-urbano), and a backup is maintained on Zenodo [6].

An outstanding feature of iNaturalist, particularly crucial for initiatives like Pomar Urbano, which demand precise taxonomic identification, is its community of identifiers. Working alongside advanced computer vision tools, this community plays a pivotal role in verifying observations to a high taxonomic resolution. Part of the platform’s success hinges on these identifiers, who constitute a small yet vital segment of iNaturalist’s user base [7]. Their expertise enhances each record’s value by refining its taxonomic classification and contributes significantly to biodiversity knowledge, especially in undersampled or ecologically significant areas [7].

## Conclusions

CS initiatives can bring forth several potential benefits to the community involved. In the case of Pomar Urbano, by actively participating, citizens can discover alternative food sources, broaden the utilization of biodiversity in their diet, enhance their connection with nature, and acquire knowledge about diverse plant species.

Monitoring engagement remains pivotal for the success of any project that relies on CS [8]. The number, quality, and frequency of user contributions can provide insights into how engaged participants are with the project. A steady or increasing number of contributions and active users indicates strong engagement and retention. iNaturalist offers tools for this purpose. The project page displays the total number of participants, enabling easy monitoring of growing contributor counts. Its subscription feature offers a more nuanced perspective, differentiating between active project subscribers and those who spontaneously add observations. Additionally, the platform bolsters enthusiasm and competition by featuring leaderboards highlighting top contributors based on observation counts.

**Figure 1:**
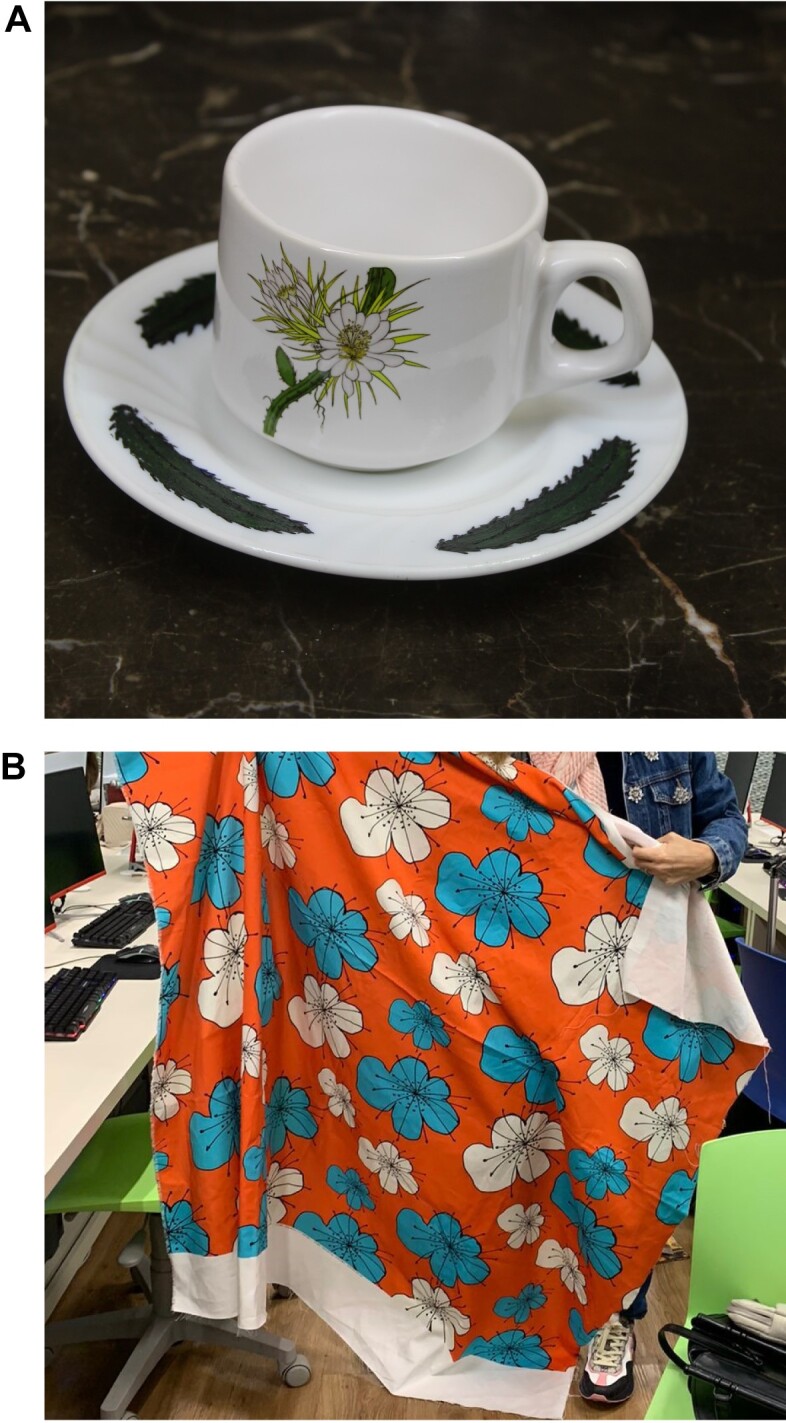
Product designs inspired by Pomar Urbano [10]. (A) A porcelain cup featuring a print inspired by the Night Blooming Cactus flower (*Epiphyllum oxypetalum*), observed during a research survey in São Paulo, Brazil. The design was created by fashion design students Kelly Cristina Soares Barbieri, Larissa Galdino de Souza Costa, and Karollina Brandão Araújo Cosso at Centro Universitário Belas Artes de São Paulo, supervised by Maria Carolina Garcia. (B) Print for a tablecloth inspired by the guava tree flower (*Psidium guajava*), using the traditional technique of Brazilian Chita. Created by Luciana Mendonca, a student of interior design at the Centro Universitário Belas Artes de São Paulo under the supervision of Maria Carolina Garcia.

In initiatives like Pomar Urbano, which focus on specific taxonomic groups, custom engagement strategies are critical. Pomar Urbano is developing a strategy for scientific dissemination to engage the Brazilian community actively. This strategy involves collaborations with social media influencers in veganism, vegetarianism, science, and environmental conservation, aiming to leverage their audiences to increase awareness and participation in Pomar Urbano. Additionally, the project has motivated professionals in the creative industry to produce unique designs inspired by the species monitored, as demonstrated in Fig. 1.

Beyond the realm of the creative industry, the reuse of CS data on urban forageable plants presents numerous opportunities. For instance, consider the project from Wageningen University and Research (WUR), which uses fruit images to train deep learning models capable of identifying irregularities in fruit quality or composition [9]. This methodology allows for detecting fraudulent activities and potential food safety concerns [9]. The success of these models hinges on the availability of a large dataset of fruit images for algorithm training. Consequently, the WUR research team is collaborating with the Pomar Urbano project to explore the feasibility of utilizing images contributed by citizen scientists for model training purposes.

For an overview of the data collected by Pomar Urbano and additional project details, please refer to the accompanying data paper by [6].

## Supplementary Material

giae007_GIGA-D-23-00261_Original_Submission

giae007_GIGA-D-23-00261_Revision_1

giae007_Response_to_Reviewer_Comments_Original_Submission

giae007_Reviewer_1_Report_Original_Submission Corey Callaghan -- 1/31/2024

## Data Availability

For access to the datasets associated with the Pomar Urbano Project please see the Data Release paper published in *GigaByte* [6].

## Abbreviations

CS: citizen science; WUR: Wageningen University and Research.
