# Supplementary material for: Leveraging citizen science for monitoring urban forageable plants
Source: Gigascience. 2024 Mar 5;13:giae007. doi: 10.1093/gigascience/giae007 (PMC10914215; doi:10.1093/gigascience/giae007)
Supplement: giae007_GIGA-D-23-00261_Original_Submission [file giae007_giga-d-23-00261_original_submission.pdf]

GigaScience

Leveraging Citizen Science for monitoring urban fruit-bearing plants

--Manuscript Draft--

**Manuscript Number:**	GIGA-D-23-00261
**Full Title:**	Leveraging Citizen Science for monitoring urban fruit-bearing plants
**Article Type:**	Commentary
**Funding Information:**	Fundação de Amparo à Pesquisa do Estado de São Paulo (21/15125-0) — Msc Filipi Miranda Soares
**Abstract:**	Urbanization brings forth social challenges in emerging countries such as Brazil, encompassing food scarcity, health deterioration, air pollution, and biodiversity loss. Despite this, urban areas like São Paulo still boast ample green spaces, offering opportunities for nature appreciation and conservation, enhancing city resilience and livability. Citizen Science is a collaborative endeavor between scientists and the society in scientific research that may help to understand the dynamics of urban ecosystems. We believe CS has the potential to promote human and nature connection in urban areas and provide useful data on urban biodiversity
**Corresponding Author:**	Filipi Miranda Soares<br>USP: Universidade de Sao Paulo<br>Sao Paulo, Sao Paulo BRAZIL
**Corresponding Author Secondary Information:**	
**Corresponding Author's Institution:**	USP: Universidade de Sao Paulo
**Corresponding Author's Secondary Institution:**	
**First Author:**	Filipi Miranda Soares
**First Author Secondary Information:**	
**Order of Authors:**	Filipi Miranda Soares
	Luís Ferreira Pires
	Maria Carolina Garcia
	Yamine Bouzembrak
	Lidio Coradin
	Natalia Pirani Ghilardi-Lopes
	Rubens Rangel Silva
	Aline Martins de Carvalho
	Benildes Coura Moreira dos Santos Maculan
	Sheina Koffler
	Uiara Bandineli Montedo
	Debora Pignatari Drucker
	Raquel Santiago
	Maria Clara Peres de Carvalho
	Ana Carolina da Silva Lima
	Hillary Dandara Elias Gabriel
	Stephanie Gabriele Mendonça de França

Powered by Editorial Manager® and ProduXion Manager® from Aries Systems Corporation

	Karoline Reis de Almeida
	Bárbara Junqueira dos Santos
	Antonio Mauro Saraiva
Order of Authors Secondary Information:	
Additional Information:	
Question	Response
Are you submitting this manuscript to a special series or article collection?	No
**Experimental design and statistics**<br><br>Full details of the experimental design and statistical methods used should be given in the Methods section, as detailed in our Minimum Standards Reporting Checklist. Information essential to interpreting the data presented should be made available in the figure legends.<br><br>Have you included all the information requested in your manuscript?	Yes
**Resources**<br><br>A description of all resources used, including antibodies, cell lines, animals and software tools, with enough information to allow them to be uniquely identified, should be included in the Methods section. Authors are strongly encouraged to cite Research Resource Identifiers (RRIDs) for antibodies, model organisms and tools, where possible.<br><br>Have you included the information requested as detailed in our Minimum Standards Reporting Checklist?	Yes
**Availability of data and materials**<br><br>All datasets and code on which the conclusions of the paper rely must be either included in your submission or deposited in publicly available repositories (where available and ethically	Yes

appropriate), referencing such data using a unique identifier in the references and in the "Availability of Data and Materials" section of your manuscript.<br><br>Have you have met the above requirement as detailed in our Minimum Standards Reporting Checklist?	

Powered by Editorial Manager® and ProduXion Manager® from Aries Systems Corporation

Click here to access/download;Manuscript;Commentary_GigaScience

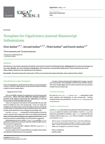

GigaScience, 2023, 1–4

doi: xx.xxxx/xxxx
Manuscript in Preparation
Commentary

COMMENTARY

Leveraging Citizen Science for monitoring urban fruit-bearing plants

Filipi Miranda Soares[1,2*], Luís Ferreira Pires[2], Maria Carolina Garcia[3], Yamine Bouzembrak[4], Lidio Coradin[5], Natalia Pirani Ghilardi-Lopes[6], Rubens Rangel Silva[7], Aline Martins de Carvalho[8], Benildes Coura Moreira dos Santos Maculan[9,14], Sheina Koffler[10], Uiara Bandineli Montedo[1], Debora Pignatari Drucker[11], Raquel Santiago[12], Maria Clara Peres de Carvalho[13], Ana Carolina da Silva Lima[14], Hillary Dandara Elias Gabriel[1], Stephanie Gabriele Mendonça de França[1], Karoline Reis de Almeida[1], Bárbara Junqueira dos Santos[15] and Antonio Mauro Saraiva[1*]

[1]Escola Politécnica, Universidade de São Paulo, São Paulo, SP, 05508-010, Brazil and [2]Faculty of Electrical Engineering, Mathematics and Computer Science, University of Twente, Enschede, 7522 NB, Netherlands and [3]Programa de Pós Graduação em Arquitetura, Urbanismo e Design, Centro Universitário Belas Artes de São Paulo, São Paulo, SP, 04018-010, Brazil and [4]Information Technology Group, Wageningen University and Research, Wageningen, The Netherlands and [5]Plants for the Future Project, Brasília, DF, 70772-090, Brazil and [6]Centro de Ciências Naturais e Humanas, Universidade Federal do ABC, São Bernardo do Campo, SP, 09606-045, Brazil and [7]Centro Universitário Una, Belo Horizonte, MG, 30160-011, Brazil and [8]Departamento de Nutrição, Faculdade de Saúde Pública, Universidade de São Paulo, São Paulo, SP, 01246-904, Brazil and [9]Programa de Pós-Graduação em Gestão & Organização do Conhecimento, Universidade Federal de Minas Gerais, Belo Horizonte, MG, 31270-901, Brazil and [10]Instituto de Estudos Avançados, Universidade de São Paulo, São Paulo, SP, 05508-060, Brazil and [11]Embrapa Agricultura Digital, Campinas, SP, 13083-886, Brazil and [12]Faculdade de Nutrição, Universidade Federal de Goiás, Goiânia, GO, 74605-080, Brazil and [13]Escola de Artes, Ciências e Humanidades, Universidade de São Paulo, São Paulo, SP, 03828-000, Brazil and [14]Escola de Ciências da Informação, Universidade Federal de Minas Gerais, Belo Horizonte, MG, 31270-901, Brazil and [15]Instituto de Pesquisas Energéticas e Nucleares, Universidade de São Paulo, São Paulo, SP, 05508-000, Brazil

[*] Correspondence authors. Filipi M. Soares. Faculty of Electrical Engineering, Mathematics and Computer Science, University of Twente, Enschede, 7522 NB, Netherlands, Phone +31 630172821. E-mail: filipisoares@usp.br, f.mirandasoares@utwente.nl and Antonio M. Saraiva. Laboratório de Automação Agrícola - Escola Politécnica da USP, Av. Prof. Luciano, Gualberto, travessa 3, nº 158, sala C2-56, Edifício de Engenharia Elétrica, Cidade Universitária - São Paulo - SP, CEP 05508-900, Fone: +55 (11) 3091-5366, Fax: +55 (11) 3091-5294, E-mail: saraiva@usp.br.

Abstract

Urbanization brings forth social challenges in emerging countries such as Brazil, encompassing food scarcity, health deterioration, air pollution, and biodiversity loss. Despite this, urban areas like São Paulo still boast ample green spaces, offering opportunities for nature appreciation and conservation, enhancing city resilience and livability. Citizen Science is a collaborative endeavor between scientists and the society in scientific research that may help to understand the dynamics of urban ecosystems. We believe CS has the potential to promote human and nature connection in urban areas and provide useful data on urban biodiversity.

Key words: Fruit-bearing plants; Native plants; Exotic plants; Biodiversity monitoring Urban biodiversity.

Background

In the dynamic landscapes of urban environments, the intricate tapestry of biodiversity is often overlooked in the midst of concrete and steel. However, an emerging force is transforming the way we perceive and comprehend the ecological fabric of cities – citizen science (CS). This commentary paper delves into the pivotal role of citizen science in monitoring urban biodiversity, unearthing its profound implications for understanding, conserving, and elevating the intricate life forms that coexist within our urban sprawls.

As urbanization continues to reshape the world, a robust understanding of the ecological dynamics within cities is indispensable for harmonizing human progress with environmental preservation. The engagement of citizen scientists, individuals with a passion for nature and an intrinsic connection to their surroundings, emerges as an ingenious solution to this challenge.

Citizen Science for urban biodiversity monitoring

CS initiatives encompass distinct levels of public participation, from collecting data to creating new research questions and projects [1]. In general, most CS projects are contributory, relying on public participation mainly for data collection. CS thus allows the creation of large datasets while approximating the public to the scientific process and providing new learning opportunities.

In the field of life sciences, especially in Ecology and Biodiversity, applications such as eBird [1], Pl@ntNet [2], and iNat [3] stand out for both their number of users worldwide and the volume of data collected. eBird and Pl@ntnet cover specific taxonomic groups, while iNat includes all forms of life.

The data available on iNat can be leveraged in monitoring urban biodiversity. While some studies have utilized iNat for this purpose (e.g., [2, 3, 4, 5, 6, 7]), there is limited research on plant diversity and distribution, such as [8]. We posit that fruit-bearing plants constitute a pivotal group of organisms crucial to the functionality of urban ecosystems, owing to their capacity to deliver an array of provisioning services. In light of this perspective, the *Pomar Urbano* (Urban Orchard) initiative serves as a collaborative platform, uniting researchers and citizen scientists across Brazil to comprehensively monitor fruit-bearing plants within urban landscapes.

iNat allows managing observations of interest within projects like *Pomar Urbano*, referred to as iNat Projects. These projects come in three types: Collection Projects, Umbrella Projects, and Traditional Projects[4]. *Pomar Urbano* is a combined collection and umbrella project. Observations posted to iNat by any user are included if they a) pertain to a plant species listed in the project and b) are located in one of the 27 Brazilian state capitals. Each capital has its own collection project. The umbrella project then aggregates data from all 27 individual collection projects. *Pomar Urbano*[5] data can be accessed via iNat, and a back-up is maintained by the project team on Zenodo.

1 https://ebird.org/home
2 https://identify.plantnet.org/
3 https://www.inaturalist.org/
4 https://www.inaturalist.org/pages/managing-projects
5 https://www.inaturalist.org/projects/pomar-urbano

Conclusions

CS initiatives can bring forth several potential benefits to the community involved. In the case of *Pomar Urbano*, by actively participating, citizens have the opportunity to discover alternative food sources, broaden the utilization of biodiversity in their diet, enhance their connection with nature, and acquire knowledge about diverse plant species.

Monitoring engagement remains pivotal for the success of any project that relies on CS [9]. The number, quality, and frequency of user contributions can provide insights into how engaged participants are with the project. A steady or increasing number of posts and active users indicates strong engagement and retention. iNat offers tools for this purpose. On the project page, it displays the total number of participants, enabling easy monitoring of growing contributor counts. Its subscription feature offers a more nuanced perspective, differentiating between active project subscribers and those whose observations are added spontaneously. Additionally, the platform bolsters enthusiasm and competition by featuring leaderboards that highlight the top contributors based on observation counts.

Projects like *Pomar Urbano* that target specific taxonomic groups require fitted engagement strategies beyond those offered by iNat. *Pomar Urbano* has devised a popular science strategy aimed at actively involving the Brazilian community. This strategic plan leverages the influence of digital creators on popular platforms like TikTok and Instagram, who specialize in vegan and vegetarian diets, science, nature, and related subjects. By partnering with these influencers, the project aims to promote *Pomar Urbano* to their engaged audience, thus fostering greater awareness and participation. Additionally, various players in the creative industry are drawing inspiration from the species monitored in the project to craft unique product designs as shown in Fig 1.

The *Pomar Urbano* initiative will reward active participants in its challenges with these products, fostering a sense of engagement and contribution. These challenges draw inspiration primarily from project-affiliated researchers who possess distinct interests in particular fruit-bearing plants. In alignment with these interests, periodic nationwide bioblitzes centered around the monitoring of specific fruit-bearing plants will be conducted.

For instance, a research team from Wageningen University & Research is engaged in harnessing fruit images to train Deep learning models that can accurately detect the geographical and production origins of fruits. To ensure the precision of these models, an extensive assemblage of fruit-specific images becomes imperative. Therefore, when a specific fruit variety, such as the banana, garners research interest, specialized challenges will be initiated with the explicit objective of amassing data tailored to that particular fruit type.

For an overview of the data gathered by *Pomar Urbano*, as well as additional project details, please refer to the Gigabyte data paper "Citizen Science Data on Human-Edible Fruit-Bearing Plants in Brazilian Urban Areas".

Declarations

List of abbreviations

AI: Artificial Intelligence; CS: Citizen Science; iNat: iNaturalist; ML: Machine Learning; GAC: Generic Artificial Consciousness; EAC: Etch a Cell; CVM: Computer Vision Model.

Compiled on: September 1, 2023.
Draft manuscript prepared by the author.

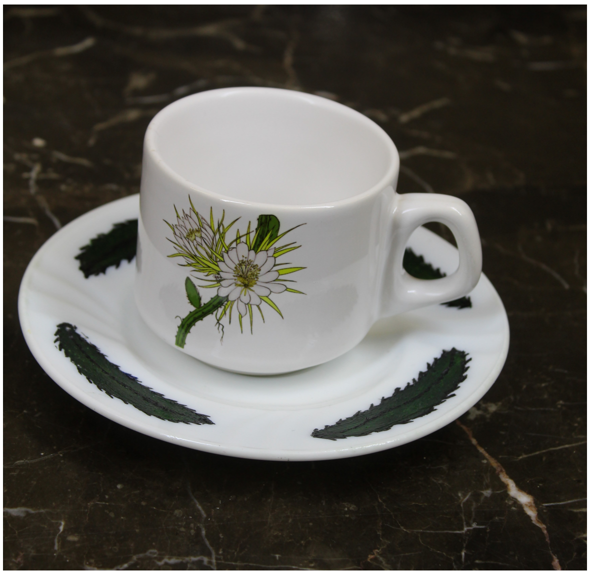

(a) A localized print on a porcelain cup inspired by the Night Blooming Cactus flower (*Epiphyllum oxypetalum*), discovered during a research survey in the city of São Paulo. Created by a group of Fashion Design students from Centro Universitário Belas Artes de São Paulo, under the supervision of Maria Carolina Garcia.

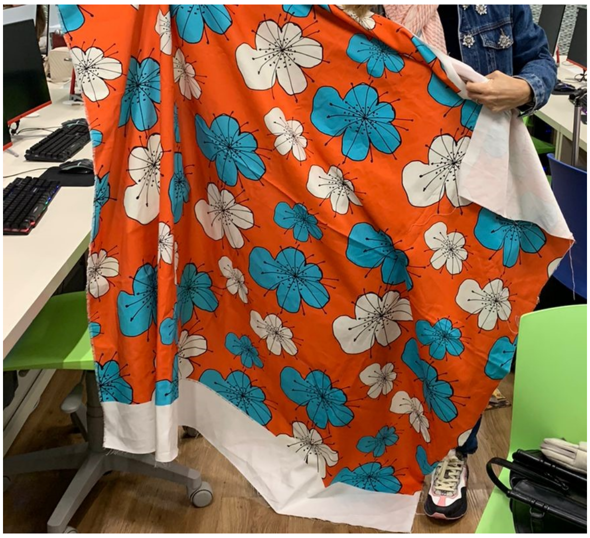

(b) Print for a tablecloth inspired by the guava tree flower (*Psidium guajava*), using the traditional technique of Brazilian Chita. Created by Luciana Mendonca, a student of Interior Design at the Centro Universitário Belas Artes de São Paulo under the supervision of Maria Carolina Garcia.

Figure 1. Product designs inspired by *Pomar Urbano* [10]

Competing Interests

Declaration of Competing Interest

The authors declare that they have no competing interest regarding the publication of this work. There are no financial, personal, or professional relationships that could be perceived as potentially biasing the content presented in this manuscript.

Author's Contributions

Study Conceptualization: FMS, MCG, AMS, LFP, BCMSM. Data curation: FMS, LC, MCPC, ACSL, SGMF, HDEG, BJS. Formal Analysis: FMS, RRS, LFP, NPGL, LC. Funding acquisition: AMS, UBM, BCMSM, MCG. Investigation: FMS, LFP, MCG, LC, NPGL, RRS, AMC, BCMSM, SK, UBM, DPD, RS, AMS. Methodology: FMS, NPGL, SK, UBM. Project administration: FMS, AMS, MCG. Supervision: AMS, LFP, BCMSM, UBM, AMC, MCG. Validation: LC. Data Visualization: FMS, RRS. Writing – original draft: FMS, LFP. Writing – review & editing: All authors made significant contributions to review, and editing of this manuscript.

Acknowledgements

We express our gratitude to the more than 2,600 citizen scientists who have made contributions through their observations. FMS thanks the Fundação de Amparo à Pesquisa do Estado de São Paulo (FAPESP) (Process number: 21/15125-0, and 22/08385-8). BCMSM thanks the Conselho Nacional de Desenvolvimento Científico e Tecnológico (CNPq) (Process number: 303650/2019-2). AMS, NPGL, SK, and FMS thank FAPESP (Process number: 2018/14994-1). SK thanks FAPESP (Process number: 2019/26760-8).

References

1. Shirk JL, Ballard HL, Wilderman CC, Phillips T, Wiggins A, Jordan R, et al. Public Participation in Scientific Research: a Framework for Deliberate Design. Ecology and Society 2012;17(2).
2. Prudic KL, Oliver JC, Brown BV, Long EC. Comparisons of Citizen Science Data-Gathering Approaches to Evaluate Urban Butterfly Diversity. Insects 2018;9(4).
3. Mape N, Alisto L, Kitching I. Hawkmoths of Baguio City, Philippines: a Preliminary Checklist Derived from Photo-based Observation Records Available on iNaturalist and Philippine Lepidoptera Internet Platforms. Philippine Journal of Science 2022 jul;151(4).
4. Vardi R, Berger-Tal O, Roll U. iNaturalist insights illuminate COVID-19 effects on large mammals in urban centers. Biological Conservation 2021;254:108953.
5. Mueller MA, Drake D, Allen ML. Using citizen science to inform urban canid management. Landscape and Urban Planning 2019;189:362–371.
6. Drake D, Dubay S, Allen ML. Evaluating human–coyote encounters in an urban landscape using citizen science. Journal of Urban Ecology 2021 01;7(1).
7. Marín-Gómez OH, Rodríguez Flores C, del Coro Arizmendi M. Assessing ecological interactions in urban areas using citizen science data: Insights from hummingbird–plant meta-networks in a tropical megacity. Urban Forestry Urban Greening 2022;74:127658.
8. Khapugin AA, Kuzmin IV, Ivanova LA. Distribution of four alien plants in Tyumen Region (Western Siberia): contribution of citizen science and expert data. Wulfenia 2021 Dec;28:151–160.
9. Golumbic YN, Baram-Tsabari A, Koichu B. Engagement and Communication Features of Scientifically Successful Citizen Science Projects. Environmental Communication

2020 May;14(4):465–480. https://www.tandfonline.com/doi/full/10.1080/17524032.2019.1687101.
10. Soares FM, Pires LF, Garcia MC, de Carvalho A, Koffler S, Ghilardi-Lopes N, et al. Optimizing the Monitoring of Urban Fruit-Bearing Flora with Citizen Science: An Overview of the Pomar Urbano Initiative. Biodiversity Information Science and Standards 2023;7.
