# Supplementary material for: Leveraging citizen science for monitoring urban forageable plants
Source: Gigascience. 2024 Mar 5;13:giae007. doi: 10.1093/gigascience/giae007 (PMC10914215; doi:10.1093/gigascience/giae007)
Supplement: giae007_GIGA-D-23-00261_Revision_1 [file giae007_giga-d-23-00261_revision_1.pdf]

GigaScience

Leveraging Citizen Science for Monitoring Urban Forageable Plants

--Manuscript Draft--

**Manuscript Number:**	GIGA-D-23-00261R1
**Full Title:**	Leveraging Citizen Science for Monitoring Urban Forageable Plants
**Article Type:**	Commentary
**Funding Information:**	Fundação de Amparo à Pesquisa do Estado de São Paulo (2021/15125-0) — Msc Filipi Miranda Soares<br>Fundação de Amparo à Pesquisa do Estado de São Paulo (2022/08385-8) — Msc Filipi Miranda Soares<br>Conselho Nacional de Desenvolvimento Científico e Tecnológico (303650/2019-2) — Dr. Benildes Coura Moreira dos Santos Maculan<br>Fundação de Amparo à Pesquisa do Estado de São Paulo (2018/14994-1) — Dr. Natalia Pirani Ghilardi-Lopes, Dr. Antonio Mauro Saraiva<br>Fundação de Amparo à Pesquisa do Estado de São Paulo (2019/26760-8) — Dr. Sheina Koffler
**Abstract:**	Urbanization brings forth social challenges in emerging countries such as Brazil, encompassing food scarcity, health deterioration, air pollution, and biodiversity loss. Despite this, urban areas like the city of São Paulo still boast ample green spaces, offering opportunities for nature appreciation and conservation, enhancing city resilience and livability. Citizen Science is a collaborative endeavor between professional scientists and non-professional scientists in scientific research that may help to understand the dynamics of urban ecosystems. We believe citizen science has the potential to promote human and nature connection in urban areas and provide useful data on urban biodiversity.
**Corresponding Author:**	Filipi Miranda Soares<br>USP: Universidade de Sao Paulo<br>Sao Paulo, Sao Paulo BRAZIL
**Corresponding Author Secondary Information:**	
**Corresponding Author's Institution:**	USP: Universidade de Sao Paulo
**Corresponding Author's Secondary Institution:**	
**First Author:**	Filipi Miranda Soares
**First Author Secondary Information:**	
**Order of Authors:**	Filipi Miranda Soares<br>Luís Ferreira Pires<br>Maria Carolina Garcia<br>Yamine Bouzembrak<br>Lidio Coradin<br>Natalia Pirani Ghilardi-Lopes<br>Rubens Rangel Silva<br>Aline Martins de Carvalho<br>Benildes Coura Moreira dos Santos Maculan<br>Sheina Koffler

Powered by Editorial Manager® and ProduXion Manager® from Aries Systems Corporation

	Uiara Bandineli Montedo
	Debora Pignatari Drucker
	Raquel Santiago
	Anand Gavai
	Maria Clara Peres de Carvalho
	Ana Carolina da Silva Lima
	Hillary Dandara Elias Gabriel
	Stephanie Gabriele Mendonça de França
	Karoline Reis de Almeida
	Bárbara Junqueira dos Santos
	Antonio Mauro Saraiva
**Order of Authors Secondary Information:**	
**Response to Reviewers:**	Dear Dr. Hans and Dr. Corey.<br>We appreciate your suggestions and insights.<br>Please see our responde to your comment, as an addition to the text:<br>Comment: "I think it is a nice commentary. Highlights the points quite well. My only comment, which is biased, would be to better highlight the important role and contributions of identifiers in the iNaturalist platform. E.g., https://doi.org/10.1371/journal.pbio.3001843."<br><br>Our answer:<br>Included in the end of Citizen Science for Urban Biodiversity Monitoring section.<br>"An outstanding feature of iNaturalist, particularly crucial for initiatives like \textit{Pomar Urbano}, which demand precise taxonomic identification, is its community of identifiers. Working alongside advanced computer vision tools, this community plays a pivotal role in verifying observations to a high taxonomic resolution. Part of the platform's success hinges on these identifiers, who constitute a small yet vital segment of iNaturalist's user base \cite{Callaghan_2022}. Their expertise enhances each record's value by refining its taxonomic classification and contributes significantly to biodiversity knowledge, especially in undersampled or ecologically significant areas \cite{Callaghan_2022}."<br><br>Please let us know what you think about this.<br>Many thanks,
**Additional Information:**	
**Question**	**Response**
Are you submitting this manuscript to a special series or article collection?	No
**Experimental design and statistics**<br><br>Full details of the experimental design and statistical methods used should be given in the Methods section, as detailed in our Minimum Standards Reporting Checklist. Information essential to interpreting the data presented should be made available in the figure legends.<br><br>Have you included all the information requested in your manuscript?	Yes

Powered by Editorial Manager® and ProduXion Manager® from Aries Systems Corporation

**Resources**<br><br>A description of all resources used, including antibodies, cell lines, animals and software tools, with enough information to allow them to be uniquely identified, should be included in the Methods section. Authors are strongly encouraged to cite Research Resource Identifiers (RRIDs) for antibodies, model organisms and tools, where possible.<br><br>Have you included the information requested as detailed in our Minimum Standards Reporting Checklist?	Yes
**Availability of data and materials**<br><br>All datasets and code on which the conclusions of the paper rely must be either included in your submission or deposited in publicly available repositories (where available and ethically appropriate), referencing such data using a unique identifier in the references and in the “Availability of Data and Materials” section of your manuscript.<br><br>Have you have met the above requirement as detailed in our Minimum Standards Reporting Checklist?	Yes

Manuscript
Click here to access/download;Manuscript;PomarUrbano_Commentary.pdf

GigaScience, 2023, 1–3

doi: xx.xxxx/xxxx
Manuscript in Preparation
Commentary

COMMENTARY

Leveraging Citizen Science for Monitoring Urban Forageable Plants

Filipi Miranda Soares[1,2*], Luís Ferreira Pires[2], Maria Carolina Garcia[3], Yamine Bouzembrak[4], Lidio Coradin[5], Natalia Pirani Ghilardi-Lopes[6], Rubens Rangel Silva[7], Aline Martins de Carvalho[8], Benildes Coura Moreira dos Santos Maculan[9,14], Sheina Koffler[10], Uiara Bandineli Montedo[1], Debora Pignatari Drucker[11], Raquel Santiago[12], Anand Gavai[13], Maria Clara Peres de Carvalho[14], Ana Carolina da Silva Lima[15], Hillary Dandara Elias Gabriel[1], Stephanie Gabriele Mendonça de França[1], Karoline Reis de Almeida[1], Bárbara Junqueira dos Santos[16] and Antonio Mauro Saraiva[1*]

[1]Escola Politécnica, Universidade de São Paulo, São Paulo, SP, 05508-010, Brazil and [2]Faculty of Electrical Engineering, Mathematics and Computer Science, University of Twente, Enschede, 7522 NB, Netherlands and [3]Programa de Pós Graduação em Arquitetura, Urbanismo e Design, Centro Universitário Belas Artes de São Paulo, São Paulo, SP, 04018-010, Brazil and [4]Information Technology Group, Wageningen University and Research, Wageningen, The Netherlands and [5]Plants for the Future Project, Brasília, DF, 70772-090, Brazil and [6]Centro de Ciências Naturais e Humanas, Universidade Federal do ABC, São Bernardo do Campo, SP, 09606-045, Brazil and [7]Centro Universitário Una, Belo Horizonte, MG, 30160-011, Brazil and [8]Departamento de Nutrição, Faculdade de Saúde Pública, Universidade de São Paulo, São Paulo, SP, 01246-904, Brazil and [9]Programa de Pós-Graduação em Gestão e Organização do Conhecimento, Universidade Federal de Minas Gerais, Belo Horizonte, MG, 31270-901, Brazil and [10]Instituto de Estudos Avançados, Universidade de São Paulo, São Paulo, SP, 05508-060, Brazil and [11]Embrapa Agricultura Digital, Campinas, SP, 13083-886, Brazil and [12]Faculdade de Nutrição, Universidade Federal de Goiás, Goiânia, GO, 74605-080, Brazil and [13]Faculty of Behavioural, Management and Social Sciences (BMS), Industrial Engineering and Business Information Systems (IEBIS) and [14]Escola de Artes, Ciências e Humanidades, Universidade de São Paulo, São Paulo, SP, 03828-000, Brazil and [15]Escola de Ciências da Informação, Universidade Federal de Minas Gerais, Belo Horizonte, MG, 31270-901, Brazil and [16]Instituto de Pesquisas Energéticas e Nucleares, Universidade de São Paulo, São Paulo, SP, 05508-000, Brazil

[*] Correspondence authors. Filipi M. Soares. Faculty of Electrical Engineering, Mathematics and Computer Science, University of Twente, Enschede, 7522 NB, Netherlands, Phone +31 630172821. E-mail: filipisoares@usp.br, f.mirandasoares@utwente.nl and Antonio M. Saraiva. Laboratório de Automação Agrícola - Escola Politécnica da USP, Av. Prof. Luciano, Gualberto, travessa 3, nº 158, sala C2-56, Edifício de Engenharia Elétrica, Cidade Universitária - São Paulo - SP, CEP 05508-900, Fone: +55 (11) 3091-5366, Fax: +55 (11) 3091-5294, E-mail: saraiva@usp.br.

Abstract

Urbanization brings forth social challenges in emerging countries such as Brazil, encompassing food scarcity, health deterioration, air pollution, and biodiversity loss. Despite this, urban areas like the city of São Paulo still boast ample green spaces, offering opportunities for nature appreciation and conservation, enhancing city resilience and livability. Citizen Science is a collaborative endeavor between professional scientists and non-professional scientists in scientific research that may help to understand the dynamics of urban ecosystems. We believe citizen science has the potential to promote human and nature connection in urban areas and provide useful data on urban biodiversity.

Key words: Fruit-bearing plants; Urban foraging; Wild food; Urban Food Trees; Food Forest; Fruit Tree.

Background

In the dynamic landscapes of urban environments, the intricate tapestry of biodiversity is often overlooked in the midst of concrete and steel. However, an emerging force is transforming the way we perceive and comprehend the ecological fabric of cities – citizen science (CS). This commentary paper delves into the pivotal role of CS in monitoring urban biodiversity, unearthing its profound implications for understanding, conserving, and elevating the intricate life forms that coexist within our urban sprawls.

As urbanization continues to reshape the world, a robust understanding of the ecological dynamics within cities is indispensable for harmonizing human progress with environmental preservation. The engagement of citizen scientists emerges as an ingenious solution to this challenge.

Citizen Science for Urban Biodiversity Monitoring

CS initiatives encompass distinct levels of public participation, from collecting data to creating new research questions and projects [1]. In general, most CS projects are contributory, relying on public participation mainly for data collection. CS thus allows the creation of large datasets while approximating the public to the scientific process and providing new learning opportunities [1].

In the field of life sciences, especially in Ecology and Biodiversity, applications such as eBird (https://ebird.org/home), Pl@ntNet (https://identify.plantnet.org), and iNaturalist (https://www.inaturalist.org/) stand out for both their number of users worldwide and the volume of data collected. eBird and Pl@ntnet cover specific taxonomic groups, while iNaturalist includes all life forms.

The data available on iNaturalist can be leveraged in monitoring urban biodiversity. While some studies have utilized iNaturalist for this purpose (e.g., [2, 3, 4]), there is limited research on plant diversity and distribution using data from this platform, such as [5]. Fruit-bearing plants constitute a pivotal group of organisms crucial to the functionality of urban ecosystems, owing to their capacity to deliver an array of provisioning services. In light of this perspective, the *Pomar Urbano* (Urban Orchard) initiative serves as a collaborative platform, uniting researchers and citizen scientists across Brazil to monitor forageable plants within urban landscapes comprehensively.

iNaturalist allows managing observations of interest within projects like *Pomar Urbano*, referred to as iNaturalist Projects. These projects come in three types: Collection Projects, Umbrella Projects, and Traditional Projects (https://www.inaturalist.org/pages/managing-projects). *Pomar Urbano* makes use of umbrella and collection projects. Observations posted to iNaturalist by any user are included if they a) pertain to a plant species listed in the project and b) are located in one of the capitals of the 27 Brazilian federative units [6]. Each capital has its collection project. The umbrella project then aggregates data from all 27 individual collection projects. *Pomar Urbano* data can be accessed via iNaturalist (https://www.inaturalist.org/projects/pomar-urbano), and a backup is maintained on Zenodo [6].

An outstanding feature of iNaturalist, particularly crucial for initiatives like *Pomar Urbano*, which demand precise taxonomic identification, is its community of identifiers. Working alongside advanced computer vision tools, this community plays a pivotal role in verifying observations to a high taxonomic resolution. Part of the platform's success hinges on these identifiers, who constitute a small yet vital segment of iNaturalist's user base [7]. Their expertise enhances each record's value by refining its taxonomic classification and contributes significantly to biodiversity knowledge, especially in undersampled or ecologically significant areas [7].

Conclusions

CS initiatives can bring forth several potential benefits to the community involved. In the case of *Pomar Urbano*, by actively participating, citizens can discover alternative food sources, broaden the utilization of biodiversity in their diet, enhance their connection with nature, and acquire knowledge about diverse plant species.

Monitoring engagement remains pivotal for the success of any project that relies on CS [8]. The number, quality, and frequency of user contributions can provide insights into how engaged participants are with the project. A steady or increasing number of posts and active users indicates strong engagement and retention. iNaturalist offers tools for this purpose. The project page displays the total number of participants, enabling easy monitoring of growing contributor counts. Its subscription feature offers a more nuanced perspective, differentiating between active project subscribers and those who spontaneously add observations. Additionally, the platform bolsters enthusiasm and competition by featuring leaderboards highlighting top contributors based on observation counts.

In initiatives like *Pomar Urbano*, which focus on specific taxonomic groups, custom engagement strategies are critical. *Pomar Urbano* is developing a strategy for scientific dissemination to engage the Brazilian community actively. This strategy involves collaborations with social media influencers in veganism, vegetarianism, science, and environmental conservation, aiming to leverage their audiences to increase awareness and participation in *Pomar Urbano*. Additionally, the project has motivated professionals in the creative industry to produce unique designs inspired by the species monitored, as demonstrated in Fig 1.

Beyond the realm of the creative industry, the reuse of CS data on urban forageable plants presents numerous opportunities. For instance, consider the project from the Wageningen University and Research (WUR), which uses fruit images to train deep learning models capable of identifying irregularities in fruit quality or composition [9]. This methodology allows for detecting fraudulent activities and potential food safety concerns [9]. The success of these models hinges on the availability of a large dataset of fruit images for algorithm training. Consequently, the WUR research team is collaborating with the *Pomar Urbano* project to explore the feasibility of utilizing images contributed by citizen scientists for model training purposes.

For an overview of the data collected by *Pomar Urbano* and additional project details, please refer to the accompanying data paper by [6].

Declarations

List of abbreviations

AI: Artificial Intelligence; CS: Citizen Science; ML: Machine Learning; GAC: Generic Artificial Consciousness; EAC: Etch a Cell; CVM: Computer Vision Model.

Competing Interests

Declaration of Competing Interest

The authors declare that they have no competing interest regarding the publication of this work. There are no financial, personal,

Compiled on: February 7, 2024.
Draft manuscript prepared by the author.

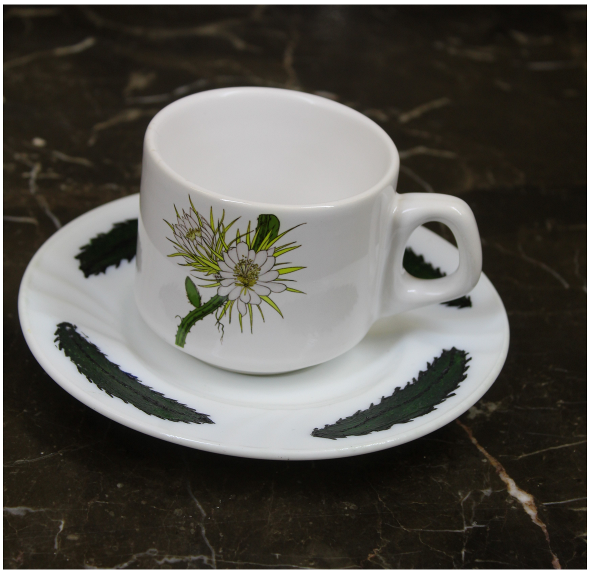

(a) A porcelain cup featuring a print inspired by the Night Blooming Cactus flower (*Epiphyllum oxypetalum*), observed during a research survey in São Paulo, Brazil. The design was created by Fashion Design students Kelly Cristina Soares Barbieri, Larissa Galdino de Souza Costa, and Karollina Brandão Araújo Cosso at Centro Universitário Belas Artes de São Paulo, supervised by Maria Carolina Garcia.

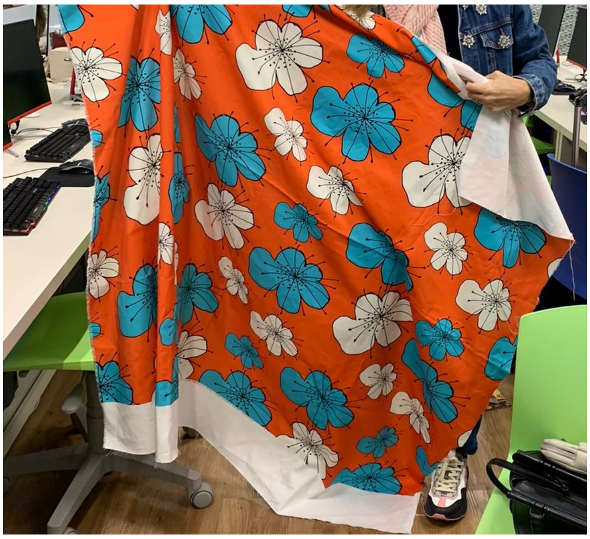

(b) Print for a tablecloth inspired by the guava tree flower (*Psidium guajava*), using the traditional technique of Brazilian Chita. Created by Luciana Mendonca, a student of Interior Design at the Centro Universitário Belas Artes de São Paulo under the supervision of Maria Carolina Garcia.

Figure 1. Product designs inspired by *Pomar Urbano* [10]

or professional relationships that could be perceived as potentially biasing the content presented in this manuscript.

Author's Contributions

Study Conceptualization: FMS, MCG, AMS, LFP, BCMSM. Data curation: FMS, LC, MCPC, ACSL, SGMF, HDEG, BJS. Formal Analysis: FMS, RRS, LFP, NPGL, LC. Funding acquisition: AMS, UBM, BCMSM, MCG. Investigation: FMS, LFP, MCG, LC, NPGL, RRS, AMC, BCMSM, SK, UBM, DPD, RS, AMS. Methodology: FMS, NPGL, SK, UBM. Project administration: FMS, AMS, MCG. Supervision: AMS, LFP, BCMSM, UBM, AMC, MCG. Validation: LC. Data Visualization: FMS, RRS. Writing – original draft: FMS, LFP. Writing – review and editing: All authors made significant contributions to review, and editing of this manuscript.

Acknowledgements

FMS thanks the Fundação de Amparo à Pesquisa do Estado de São Paulo (FAPESP) (Process number: 21/15125-0, and 22/08385-8). BCMSM thanks the Conselho Nacional de Desenvolvimento Científico e Tecnológico (CNPq) (Process number: 303650/2019-2). AMS, NPGL, SK, and FMS thank FAPESP (Process number: 2018/14994-1). SK thanks FAPESP (Process number: 2019/26760-8).

References

1. Shirk JL, Ballard HL, Wilderman CC, Phillips T, Wiggins A, Jordan R, et al. Public Participation in Scientific Research: a Framework for Deliberate Design. Ecology and Society 2012;17(2).
2. Prudic KL, Oliver JC, Brown BV, Long EC. Comparisons of Citizen Science Data-Gathering Approaches to Evaluate Urban Butterfly Diversity. Insects 2018;9(4).
3. Drake D, Dubay S, Allen ML. Evaluating human–coyote encounters in an urban landscape using citizen science. Journal of Urban Ecology 2021 01;7(1).
4. Marín-Gómez OH, Rodríguez Flores C, del Coro Arizmendi M. Assessing ecological interactions in urban areas using citizen science data: Insights from hummingbird–plant meta-networks in a tropical megacity. Urban Forestry & Urban Greening 2022;74:127658.
5. Khapugin AA, Kuzmin IV, Ivanova LA. Distribution of four alien plants in Tyumen Region (Western Siberia): contribution of citizen science and expert data. Wulfenia 2021 Dec;28:151–160.
6. Soares FM, Pires LF, Garcia MC, Coradin L, Ghilardi-Lopes NP, Silva RR, et al. Citizen Science Data on Urban Forageable Plants: A Case Study in Brazil. GigaByte 2024;doi: 10.46471/gigabyte.107.
7. Callaghan CT, Mesaglio T, Ascher JS, Brooks TM, Cabras AA, Chandler M, et al. The benefits of contributing to the citizen science platform iNaturalist as an identifier. PLOS Biology 2022 Nov;20(11):e3001843.
8. Golumbic YN, Oesterheld M. From goals to engagement—evaluating citizen science project descriptions as science communication texts. Frontiers in Environmental Science 2023 Sep;11:1228480.
9. Marvin HJ, Hoenderdaal W, Gavai AK, Mu W, van den Bulk LM, Liu N, et al. Global media as an early warning tool for food fraud; an assessment of MedISys-FF. Food Control 2022;137:108961.
10. Soares F, Ferreira Pires L, Garcia MC, De Carvalho A, Koffler S, Ghilardi-Lopes N, et al. Optimizing the Monitoring of Urban Fruit-Bearing Flora with Citizen Science: An Overview of the Pomar Urbano Initiative. Biodiversity Information Science and Standards 2023 Sep;7:e112009.
