# Supplementary material for: Leveraging citizen science for monitoring urban forageable plants
Source: Gigascience. 2024 Mar 5;13:giae007. doi: 10.1093/gigascience/giae007 (PMC10914215; doi:10.1093/gigascience/giae007)
Supplement: giae007_Response_to_Reviewer_Comments_Original_Submission [file giae007_response_to_reviewer_comments_original_submission.pdf]

Author's Response To Reviewer Comments

Dear Dr. Hans and Dr. Corey.
We appreciate your suggestions and insights.
Please see our responde to your comment, as an addition to the text:
Comment: "I think it is a nice commentary. Highlights the points quite well. My only comment, which is biased, would be to better highlight the important role and contributions of identifiers in the iNaturalist platform. E.g., https://doi.org/10.1371/journal.pbio.3001843."

Our answer:
Included in the end of Citizen Science for Urban Biodiversity Monitoring section.
"An outstanding feature of iNaturalist, particularly crucial for initiatives like \textit{Pomar Urbano}, which demand precise taxonomic identification, is its community of identifiers. Working alongside advanced computer vision tools, this community plays a pivotal role in verifying observations to a high taxonomic resolution. Part of the platform's success hinges on these identifiers, who constitute a small yet vital segment of iNaturalist's user base \cite{Callaghan_2022}. Their expertise enhances each record's value by refining its taxonomic classification and contributes significantly to biodiversity knowledge, especially in undersampled or ecologically significant areas \cite{Callaghan_2022}."

Please let us know what you think about this.
Many thanks,
